# Empathic responses to unknown others are modulated by shared behavioural traits

**DOI:** 10.1038/s41598-020-57711-6

**Published:** 2020-02-06

**Authors:** Silke Anders, Christian Beck, Martin Domin, Martin Lotze

**Affiliations:** 1 Social and Affective Neuroscience, Department of Neurology, Universität zu Lübeck, Lübeck, Germany; 2 Functional Imaging Unit, Center for Diagnostic Radiology, University Medicine Greifswald, Greifswald, Germany

**Keywords:** Social evolution, Empathy

## Abstract

How empathically people respond to a stranger’s pain or pleasure does not only depend on the situational context, individual traits and intentions, but also on interindividual factors. Here we ask whether empathic responses towards unknown others are modulated by behavioural similarity as a potential marker of genetic relatedness. Participants watched two supposed human players who were modelled as having a strong (player LP) or weak (player NLP) tendency to lead in social situations executing penalty shots in a virtual reality robot soccer game. As predicted, empathic response were modulated by shared behavioural traits: participants whose tendency to lead was more similar to player LP’s tendency to lead experienced more reward, and showed stronger neural activity in reward-related brain regions, when they saw player LP score a goal, and participants whose tendency to lead was more similar to player NLP’s tendency to lead showed stronger empathic responses when they saw player NLP score a goal. These findings highlight the potentially evolutionary grounded role of phenotypic similarity for neural processes underlying human social perception.

## Introduction

Complex modern societies require individuals to interact benevolently not only with personally acquainted others but also with complete strangers. How readily individuals respond empathically to the needs and desires of their fellow human beings does not only depend on the situational context and individual traits and intentions, but also on interindividual factors. According to David Kenny^1^, any response to another person can be decomposed into at least four components: a context effect, a perceiver effect, a target effect and a target-perceiver effect. Importantly, significant target-perceiver effects can arise not only between personally known others, but also between strangers.

Sociobiological accounts of human social behaviour surmise that selfless (altruistic) social behaviour has emerged through kin selection^2–4^. According to kin selection theories individuals should not only act selflessly towards their close kin, but also unfamiliar others if they share a sufficiently large proportion of their genes^5^. Genetic relatedness to unfamiliar others is thought to be signalled through phenotypic similarity: If I help someone who is similar to me the chance is high that I help someone who shares some of my genes (“phenotype matching”^6^). However, currently there is little evidence that human empathic responses towards unknown others are modulated by phenotypical similarity between target and perceiver.

Neuroimaging studies have examined whether humans show stronger empathic pain responses towards another person’s pain when they perceive the other person as belonging to their own ethnicity than when they perceive the other person as belonging to a different ethnicity (signalled by similar/dissimilar facial physiognomy and/or skin colour). Although participants in these studies typically showed stronger pain-related neural responses towards targets of their own ethnicity than towards targets of a different ethnicity it remains unclear whether this response bias was due to innate behavioural tendencies or whether it arose from learned (culturally acquired) preferences towards members of one’s own ethnicity^7–10^.

Using a different approach, Mobbs and colleagues^11^ investigated whether people experience more empathic reward when they see another person succeed who they perceive as sharing their own attitudes than when they see another person succeed who they perceive as having dissimilar attitudes. Perceived similarity was manipulated by the use of pre-recorded videos in which two supposed contestants verbally expressed either socially desirable or socially undesirable attitudes on social and ethical issues. After seeing the videos, participants watched the two supposed contestants betting on cards. Participants reported to experience more empathic reward, and showed stronger neural activity in reward-related brain regions, when they saw the contestant win who they rated as more similar to themselves than when they saw the other contestant win.

This study provided first evidence that empathic responses might be modulated by perceived attitudinal similarity. However, it also leaves some key questions unresolved. First, verbally expressed attitudes often reflect desirable rather than actual individual traits^12,13^. Thus, phenotypic similarity inferred from a target’s verbal statements might not constitute a valid cue to genetic relatedness for the perceiver, questioning whether the observed modulation of empathic responses indeed reflected kin selection mechanisms. Second, due to the way similarity was manipulated (supposed contestants were modelled as having either socially desirable or socially undesirable attitudes), similarity ratings were significantly higher for one (the desirable) than for the other (the undesirable) target across all participants, making it difficult to disentangle the roles of social desirability and similarity. Finally, target-perceiver similarity was assessed by self-report, which might be skewed by a perceiver’s tendency to see them as being more similar to the target modelled as having socially desirable traits^12,13^ (and might thus not reflect actual similarity).

**Figure 1:**
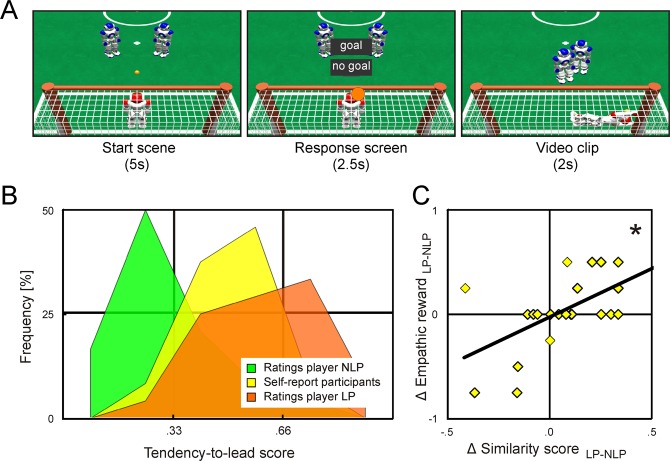
(**A**) Timeline of a robot soccer trial. Trials comprised a *start scene*, showing the two robots standing side-by-side at the penalty spot, a *response screen*, indicating the side of the goal the goalie would block by an orange disc and asking the participant to enter their response by button press, and the *video clip* showing one of the robots executing the shot, the goalie blocking the goal, and the shot resulting in a goal or no goal. (**B**) Frequency distribution of the participants’ ratings of the supposed players’ tendency to lead and their own tendency to lead (LP, player who supposedly executed 75 of the shots; NLP, player who supposedly executed 25 percent of the shots). (**C**) Correlation between individual differences in behavioural similarity and individual differences in experienced empathic reward. Each dot denotes a participant. The asterisk indicates a significant effect (p < 0.05).

The aim of the current study was to examine whether empathic responses towards unkonwn others are modulated by the degree to which target and perceiver share behavioural traits that can be inferred from the target’s non-verbal behaviour (i.e., without relying on the target’s self-report) and that are not strongly valued by social norms (i.e., that are not generally perceived as being desirable or undesirable). For this, we used an experimental approach similar to that used by Mobbs and colleagues^11^, except our the targets differed in a non-verbal behavioural pattern that (in Western societies) is not strongly valued by social norms (i.e., the tendency to take the lead in social situations). Participants were told that they would see video clips of two human players interacting in a virtual reality robot soccer penalty shoot out (by moving two humanoid robots), and that the common goal of the players was to defeat the soft-ware driven goalie and to score as many goals as possible. Unbeknownst to the participants, the supposed players were modelled to differ in how often they took the lead and executed a penalty shot: One player (LP) was modelled as having a strong tendency to lead and executed 75 percent of the shots, and the other player (NLP) was modelled as having a weak tendency to lead and executed 25 percent of the shots (Fig. 1A). All other aspects of the supposed players’ behaviour (including their relative success in converting shots into goals) were kept similar. Participants were shown a series of penalty shoot outs while their brain activity was recorded with fMRI (functional magnetic resonance imaging). To keep the participants’ attention focussed on the players’ behaviour they were asked to predict the players’ behaviour in each trial by button press. After imaging, participants completed two questionnaires, one assessing their own trait tendency to lead in social situations and the other assessing their perception of the two supposed players. These ratings were used to compute a similarity score for each participant and player that reflected the participant’s and the player’s similarity with regard to their tendency to lead in social situations.

We predicted that (a) the degree of self-reported empathic reward and (b) the strength of neural responses in reward-related brain regions elicited when a participant saw a player successfully converting a shot into a goal (versus when they saw the player executing a shot that was not converted into a goal) would be predicted by shared behavioural traits: the more similar a participant’s self-reported tendency to lead was to one player’s tendency to lead (relative to the other player’s tendency to lead), the stronger the participant’s empathic response towards this player (relative to the other player) should be.

## Results

### Behavioural results – target perception

Button presses recorded during fMRI indicated that participants quickly learned that the two supposed players (LP and NLP) differed in how often they took the lead and executed a penalty shot (Fig. S1 in the Supplemental Material), and post-scan ratings showed that more than 80 percent of participants perceived player LP as having a higher tendency to lead than player NLP (*tendency-to-lead*_LP_ = 0.61 +/− 0.04 [mean +/−  s.e.m.], *tendency-to-lead*_NLP_ = 0.25 +/− 0.03, Δ *tendency-to-lead*_LP-NLP_ = 0.36 +/− 0.06, Cohen’s d = 1.2; T[23] = 5.7, p < 0.001, Fig. 1B**)**. In contrast, participants disagreed on which player they found more likable, and for which player they felt more empathic reward. About 40 percent of participants rated player LP as more likable than player NLP, and about 25 percent rated player NLP as more likable than player LP (Δ *liking*_LP-NLP = _0.07 +/− 0.08, Cohen’s d = 0.20; T[23] = 0.9, p > 0.200), indicating that participants did not generally prefer one behavioural pattern over the other. About 33 percent of participants reported to have experienced more reward when they saw player LP score a goal, and about 15 percent reported to have experienced more reward when they saw player NLP score a goal (Δ *empathic reward*_LP-NLP = _0.04 +/− 0.07, Cohen’s d = 0.1; T[23] = 0.6, p > 0.200), indicating that participants also differed in whose players’ success they shared more empathically.

### Behavioural results – target-perceiver similarity

Overall, participants rated their tendency to lead as slightly weaker than player LP’s tendency to lead and as stronger than player NLP’s tendency to lead (*tendency-to-lead*_participants_ = 0.51 +/−  0.03, Fig. 1B). Importantly, self-reported tendency-to-lead scores of about 66 percent of participants were more similar to their tendency-to-lead ratings for player LP, and self-reported tendency-to-lead scores of about 33 percent of participants were more similar to their tendency-to-lead ratings for player NLP. Similarity scores for the two players ([1 – |t*endency-to-lead*_participant_ - *tendency-to-lead*_player_|], see **Methods**) ranged from 0.36 to 1 (on a scale from 0 to 1). On average, similarity scores were slightly higher for player LP than for player NLP, but this effect was small and statistically not significant (*similarity score*_LP_ = 0.80 +/− 0.03; *similarity score*_NLP_ = 0.72 +/− 0.03, Δ *similarity*_LP-NLP_ = 0.08 +/− 0.05; Cohen’s d = 0.33; T[23] = 1.6, p = 0.124).

### Behavioural results – target-perceiver similarity and empathic reward

Supporting the first part of our hypothesis (a), across participants individual differences in self-reported empathic reward for player LP and player NLP (Δ *empathic reward*_LP-NLP_) were explained by individual differences in behavioural similarity to player LP and Player NLP (Δ *similarity*_LP-NLP_) (r = 0.61, T[22] = 3.6, p = 0.002, Fig. 1C). This correlation remained significant when differences in liking (Δ *liking*_LP-NLP_) were removed from both variables (r = 0.53, T[21] = 2.9, p = 0.008).

### Neuroimaging results – own reward

**Figure 2:**
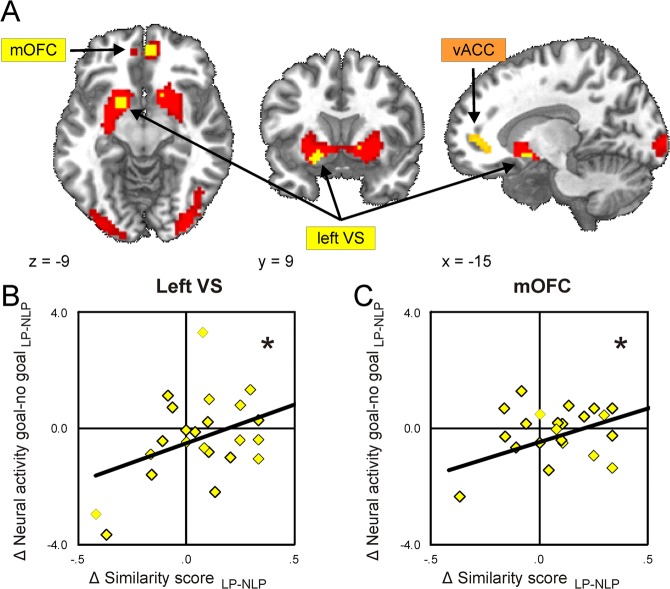
Neural activity associated with own reward and empathic reward. (**A)** Neural activity associated with own reward (red, Δ *neural activity correct-incorrect response*) in the VS/mOFC and neural activity modulated by behavioural similarity in the vACC (orange, Δ *similarity*_LP-NLP_ x Δ *neural activity goal-no goal*_LP-NLP_). Statistical parametric maps are thresholded at a voxel-wise height threshold of p = 0.001 and superimposed onto sections of a T1-weighted map of a standard brain (MNI). Functionally defined ROIs in the VS and mOFC (from which data in **B**,**C** were extracted) are shown in yellow. (**B,C**) Correlation between differences in behavioural similarity and differences in empathic-reward related neural activity in the left VS and mOFC. Each dot denotes a participant. Asterisks indicate significant effects (p < 0.05). *LP*, player who supposedly executed 75% of the shots; *NLP*, player who supposedly executed 25% of the shots. *VS*, ventral striatum, *mOFC*, medial orbitofrontal cortex, *vACC*, ventral anterior cingulate cortex.

In line with the literature^14,15^ neural activity in a large cluster comprising left and right ventral striatum (VS) and in a second large cluster in the medial orbitofrontal cortex (mOFC) increased when a participant saw that they had correctly predicted the outcome of a trial (relative to when they saw that they had not correctly predicted the outcome) (left VS, x = −18, y = 9, z = −12, T[23] = 10.7; right VS, x = 15, y = 15, z = −3, T[23] = 9.5; k = 1069 voxels, p < 0.001; mOFC, x = 6, y = 51, z = −6, T[23] = 5.3, k = 313 voxels, p < 0.001; all p FWE corrected at cluster level, Table S2 in the Supplemental Material). Additionally, we observed an unpredicted increase of neural activity in this contrast in bilateral occipital cortex (left occipital cortex, x = −15, y = −102, z = 3, T[23] = 5.2, k = 350 voxels; right occipital cortex, x = 33, y = −93, z = −6, T[23] = 6.2, k = 292 voxels; p < 0.001; p FWE corrected at cluster level, Fig. 2A, Table S2 in the Supplemental Material).

### Neuroimaging results – target-perceiver similarity and empathic reward

To examine whether differences in similarity scores predicted differences in neural responses in reward-related brain regions when a participant saw a player successfully score a goal (versus when they saw that player executing a shot that was not converted into a goal) we extracted contrast estimates from three spherical ROIs (regions of interest) centred at the most significantly activated voxels in the left and right VS and mOFC, respectively, in the analysis of own reward. We then tested whether differences in similarity scores (Δ *similarity*_LP-NLP_) predicted differences in empathic-reward related neural activity (Δ *neural activity goal-no goal*_LP-NLP_) in these ROIs. This was the case in the left VS and mOFC (Δ *similarity*_LP-NLP_ x Δ *neural activity goal-no goal*_LP-NLP_, left VS, r = 0.43, T[22] = 2.2, p = 0.039; mOFC, r = 0.45, T[22] = 2.4, p = 0.025, Fig. 2B,C), but not in the right VS (r = 0.26, T[22] = 1.3, p > 0.200). Importantly, correlations in the left VS dropped just below statistical significance, and correlations in the mOFC remained significant, when individual differences in liking (Δ *liking*_LP-NLP_) were removed (left VS, r = 0.37, T[22] = 1.9, p = 0.071; mOFC, r = 0.51, T[22] = 2.8, p = 0.010). Thus, in line with the second part of our hypothesis (b), individual differences in behavioural similarity predicted individual differences in empathic-reward related neural activity in the left VS and mOFC (see Fig. S2 in the Supplemental Material for separate correlation analyses for each player and Fig. S3 in the Supplemental Material for ROI-based voxel-wise correlation analyses**)**.

### Neuroimaging results – target-perceiver similarity and neural activity outside VS/mOFC

A post-hoc whole brain analysis, searching for brain regions outside the VS/mOFC whose activity would be modulated by differences in behavioural similarity revealed a cluster in the ventral anterior cingulate cortex (vACC) (Δ *similarity*_LP-NLP_ x Δ *neural activity goal-no goal*_LP-NLP_, vACC, x = −12, y = 48, z = 3, T[22] = 5.3, k = 109 voxels, p = 0.005, p FWE- corrected at cluster level, Fig. 3A, Table S2 in the Supplemental Material). Post-hoc functional connectivity analyses revealed that differences in neural activity in this region (mean contrast estimates in a spherical ROI as in the VS/mOFC ROI analyses above) covaried with differences in neural activity in the left VS and mOFC across participants (Δ *vACC goal-no goal*_LP-NLP_ x Δ *left VS goal-no goal*_LP-NLP_, r = 0.42, T[22] = 2.2, p = 0.042; Δ *vACC goal-no goal*_LP-NLP_ x Δ *mOFC goal-no goal*_LP-NLP_, r = 0.59, T[22] = 3.4, p = 0.001), suggesting a role of this region for detecting and/or representing self-similarity. No correlation between individual differences in behavioural similarity and individual differences in neural responses to a player’s failure (Δ *similarity*_LP-NLP_ x Δ *neural activity no goal-goal*_LP-NLP_) were observed, neither at a conventional voxel-wise height threshold of p = 0.001 (no extent threshold), nor when the voxel-wise height threshold was lowered to p = 0.010 (using an extent threshold of k = 10 voxels).

### Supplemental study

The current study aimed to test whether empathic responses towards unknown others are modulated by behavioural similarity. For this we sought to implement a measure of similarity that did not require participants to directly rate self-similarity, because self-ratings might be skewed by social demands^12,13^ and, if obtained after behavioural observation, might be affected by empathic-reward related neural activity that might occur during behavioural observation (i.e. participants might tend to rate targets as more similar to themselves for whom they had experienced more empathic reward during behavioural observation). Thus, we used a behavioural similarity measure that was based on two independent questionnaires that did not directly assess perceived similarity.

To ensure the external validity of this measure we performed a supplemental study with two groups of participants in which participants (i) interacted with one another, (ii) rated how similar each participant was to themselves, and (iii) completed the two tendency-to-lead questionnaires used to compute similarity scores in the main study. Analysis of these data showed that a significant amount of variance in self-rated similarity was explained by similarity scores computed from the two questionnaires (Fig. S4 in the Supplemental Material). This demonstrates (i) that when people assess how similar another person is to themselves, perceived tendency-to-lead is an important factor (see Supplemental Material for further discussion) and (ii) the similarity score computed in the current study provided a valid estimate of perceived self-similarity.

## Discussion

Observational studies suggest that human selfless behaviour is modulated by genetic relatedness^16,17^, but there has been little evidence that human altruistic responses towards others increase with phenotypic similarity as proposed by kin-selection theories of human social behaviour. The current study examined whether empathic responses, an important precursor of altruistic behaviour^18–21^, towards unknown others are modulated by shared behavioural traits inferred from the other person’s non-verbal behaviour. Participants were asked to observe two supposed human players who were modelled as having either a strong (player LP) or a weak (player NLP) tendency to lead in social situations executing penalty shots in a virtual reality robot soccer game. As predicted, we found a significant three-way interaction: participants whose self-reported tendency to lead was more similar to player LP’s tendency to lead experienced more reward, and showed stronger neural activity in reward-related brain regions, when they saw player LP score a goal than when they saw player NLP score a goal, and *vice versa*. These findings suggest that empathic responses towards unknown others are modulated by behavioural similarity, and partly support with kin-selection theories of human social behaviour^3–5^.

Importantly, the modulation of empathic responses observed in the current study cannot easily be explained by learned preferences or social norms that might value some behavioural traits higher than others. First, although the vast majority of participants perceived player LP as having a stronger tendency to lead than player NLP, participants disagreed on which player was more likable. Second, participants had very similar socio-cultural backgrounds but showed different neural responses when they saw one or the other player score a goal. Third, similarity scores were computed based on two independent questionnaires that did not directly assess perceived similarity, making them robust against biases that can be induced by social demands^12,13^. Thus, the current study extends previous studies that observed significant phenotype-dependent target-perceiver effects in neural responses towards others’ pain^7–10^ or pleasure^11^ by showing that phenotype-dependent modulation of empathic responses is not restricted to physical^7–10^ or attitudinal^11^ similarity but can be induced by similarity of behavioural traits that are not strongly valued by social norms.

Participants in the current study showed significant phenotype-dependent modulation of self-reported empathy and neural responses in left VS and mOFC, two brain regions that also responded when participants saw that they had correctly predicted the outcome of a trial and that are regarded core regions of the brain’s reward system^14,15^. In addition to these predicted effects we observed a significant phenotype-dependent modulation of neural activity in a cluster in the ventral anterior cingulate cortex (vACC) which was linked to the modulation of neural activity in the left VS and mOFC. The vACC is part of a larger brain region that has been found to play an important role in representing others in relation to oneself, the ventromedial prefrontal cortex (vmPFC)^22.^ Specifically, activity in a region of the vmPFC that partly overlaps with the cluster in the vACC detected in the current study has been shown to increase when people make judgments about others who they believe share their own preferences and attitudes (compared to when they make similar judgments about others they perceive as dissimilar to themselves)^23–25^. Furthermore, greater attitudinal similarity was associated with stronger functional coupling between the vACC and VS in the study by Mobbs and colleagues^11^. Together, these findings point towards a potential role of the vACC/vmPFC for detecting and/or representing self-similarity and modulating prosocial responses accordingly.

Significant target-perceiver effects have also been observed in studies examining in-group/out-group effects in empathic pain and pleasure. In these studies participants typically show stronger responses to another person’s pain or pleasure when they believe that this person to their own social group than when they believe that the person belongs to a different social group^26–28^. Interestingly, the mPFC also seems to play a role in mediating these effects^29,30^. This suggests that similarity-induced target-perceiver effects and in-group/out-group effects might be mediated by similar neural mechanisms, even though sociobiological theories assume that they might have emerged through different evolutionary processes (e.g^2^.). Clearly, more research is needed to better understand the neural processes underlying interindividual effects in social responses towards unfamiliar others and how they translate to related concepts such as social proximity and distance (e.g.^31^), interindividual attraction (e.g.^32^), feelings of affection (e.g.^33,34^) and social bonds (e.g.^35,36^). Integrative studies examining different factors with larger sample sizes than the current and previous studies^7–11^ are needed to gain a deeper insight into the neural processes that mediate target-perceiver effects in human social perception and behaviour.

In sum, the findings of the current study show that significant phenotype-dependent interindividual effects between unfamiliar others can arise from differences in behaviour that are not strongly valued by social norms. We believe that a deeper understanding of the role of such potentially genetically grounded interindividual effects in empathic responses towards others will not only enhance our understanding of the neural processes that govern human social perception, but might also affect our view on human social behaviour in a rapidly changing and increasingly anonymous social world.

## Methods

### Participants

Twenty-nine participants with no record of neurological or psychiatric disorders (assessed by self-report) were recruited at the University of Greifswald, Germany. Data of two participants were excluded because these participants did not believe in the cover story or did not use all response options in all conditions, and data of three other participants were excluded because of significant head movement during fMRI (>3 mm within a run or >10 mm between runs). The final data set included data from 24 participants (13 women, 11 men, mean age 24.6 years, range 19–34 years). The study was conducted in accordance with the *Declaration of Helsinki* and participants gave written informed consent before participation. The study was approved by the Ethics committee of the Universität zu Lübeck, Germany.

### Experimental procedure

Participants were told that during fMRI they would see a series of pre-recorded video clips showing two humanoid robots that were driven by two human players (participants in a previous study) interacting in a virtual robot soccer penalty shoot-out. Participants were further told that the human players were team mates who had the common goal to defeat the software-driven goalie and to score as many goals as possible. To keep the participants’ attention focussed on the supposed players’ behaviour, and to obtain data to localize reward-related neural activity in the participants’ brain, participants were told that their task would be to predict the players’ behaviour in each trial more accurately than the software that supposedly controlled the goalie.

Each trial commenced with a start scene (5 s) showing the two humanoid robots standing side-by-side at the penalty spot (Fig. 1A). Robots were marked by different eye and chest mark colours (orange and green, respectively) and assignment of the robots to players was counterbalanced across participants). The start scene was followed by a response screen (2.5 s) indicating the side of the goal the soft-ware driven goalie would block by an orange disc and asking the participant to enter their response. After these two still pictures participants were shown the full video clip (2 s) showing the robots executing the shot, the goalie blocking the goal, and the shot resulting in a goal or no goal (trial types are listed in Table S1 in the Supplemental Material). Trials were separated by a baseline (15.5 s) with a white cross hair on a black screen. Because shots could only be executed into the far corner of the goal, predicting whether a shot would result in a goal or no goal in a given trial was equivalent to predicting which player would execute the shot in that trial (see Table S1). Thus, the participants’ in-scan responses could be used to assess the participants’ prediction of the players’ behaviour on a trial-by-trial basis.

To familiarize participants with their task and the robots’ appearance they completed a number of practise trials in the scanner (with robots with eye and chest mark colours not used in the main experiment) and passively viewed a series of portrait pictures [1.5 s] of the two robots used in the main experiment (18 pictures per robot) before the main experiment started. The series of portrait pictures was shown once again after the main experiment. FMRI data of the pre- and post portrait picture runs were not used in the current study. Stimulus presentation and response logging were controlled with the Psychophysics toolbox (PTB-3, http://psychtoolbox.org)^37–39^ in Matlab (version R2011a, TheMathWorks, Natick, MA, USA).

### Manipulation of the robots’ behaviour

Four different series of 128 pseudorandomized video clips (randomly assigned to different participants) were compiled with the restriction that each of the 16 possible configurations of start position and block direction in two consecutive trials occurred once within overlapping blocks of 16 trials (8 trials overlap). A simple rule determined which robot executed the shot in each trial: The robot assigned to player LP executed the shot whenever its start position was on the right side of the penalty spot (50% of all trials, Table S1), and, additionally, when its start position was on the left side and the previous trial had resulted in a goal (25% of all trials), the robot assigned to player NLP executed the shot whenever its start position was on the left side and the previous trial had not resulted in a goal (25% of all trials). The robots’ behaviour was modelled to follow a rule (rather than a random distribution) in order to keep participants engaged in predicting the players’ behaviour. Block directions were balanced over the shooters’ start position, resulting in 50% successfully converted shots (goals) and 50% not successfully converted shots (no goals) for each robot. Robot soccer trials were created with *Webots* 6.3.2 (http://www.cyberbotics.com) using simulated *NAO* robots 3.0 (https://www.aldebaran.com) and robot-behaviour assignments were counterbalanced across participants.

### MRI data acquisition

MRI data were obtained on a 3 Tesla scanner equipped with a 32 channel head coil at the University of Greifswald, Greifswald, Germany (Siemens Verio, Germany). Functional imaging was divided into 4 runs (320 scans per run). Functional data were acquired with a single-shot gradient echo T2*-weighted EPI sequence covering the whole brain (40 axial slices, slice thickness 2.5 mm + 0.5 mm gap, interleaved order, in-plane resolution 2.5 × 2.5 mm², FOV 220 × 220 mm², flip angle 70°, TE = 30 ms, TR = 2500 ms, GRAPPA, iPAT = 2). To allow for T1 saturation each run was preceded by acquisition of five scans which were not included in the analysis. A T1-weighted anatomical image (MPRAGE, 160 sagital slices, resolution 1 × 1 × 1 mm³, FOV 240 × 240 mm²), used to register functional data into MNI space^40^, and a T2-weighted gradient echo image (same geometry as for the functional data, TR = 488 ms, TE_1 = _4.92 ms, TE_2 = _7.38 ms, flip angle 60°), used to correct for image distortions, were obtained from each participant before functional imaging.

### Post-scan ratings

After imaging participants completed two questionnaires. The first questionnaire comprised five questions relating to the participant’s perception of each of the two supposed players, placed next to a picture of the respective player’s robot. The first two items assessed how likeable the participant found the supposed player and how much vicarious reward they had experienced when they saw that player score a goal. The other three items related to the supposed player’s tendency to lead (assertiveness-influence-aggressiveness). Ratings for the last three items were averaged (separately for each participant) to a tendency-to-lead score for each supposed player. The second questionnaire comprised 23 self-report items taken from the *Assertiveness* and *Dominance* scales of the *International Personality Item Pool*^41^ (IPIP, http://ipip.ori.org/newCPIKey.htm) and assessed the participants’ own tendency to lead in social situations. All rating were obtained on 5-point scales ranging from [1] (not at all) to [5] (very much) and were linearly transformed to range from [0] to [1] for further analysis and visualization. Individual similarity scores were computed for each participant and player by computing the absolute difference between the participant’s rating of the player’s tendency to lead (assertiveness-influence-aggressiveness scales) and their rating of their own tendency to lead (IPIP), and contrasting this difference with 1 ([1 – |*tendency-to-lead*_participant_ - *tendency-to-lead*_player_|]). For all measures, individual difference scores for player LP and NLP were obtained by subtracting individual scores for player LP with individual scores for player NLP.

### MRI data analysis

MRI data were analyzed with SPM8 (Wellcome Department of Imaging Neuroscience, University College London, London, UK) using Matlab (version R2011a, TheMathWorks, Natick, MA, USA). Preprocessing followed standard procedures and included (i) concurrent spatial realignment and correction of image distortions, (ii) normalization into standard MNI space (using individual T1-weighted images of each participant, the SPM8 T1-weighted template and DARTEL), and (iii) resampling at a spatial resolution of 3 × 3 × 3 mm³. BOLD (blood oxygen level dependent) responses during each trial were estimated using a GLM for each participant that accounted for first-order autocorrelations and low-frequency drifts (high-pass cut-off 128 s). Each GLM included three regressors per trial (box car functions convolved with the canonical hemodynamic response function), modelling the three parts of each trial (*start scene* [5 s] – *response screen* [2.5 s] – *video clip* [2 s]). All contrasts of interest (see below) were based on parameter estimates for *video clips* (showing the shot being executed).

Two contrasts of interest were computed for each participant: Δ *neural activity correct-incorrect response* (*video clips* in which the participant saw that they had correctly predicted the outcome of the trial contrasted with *video clips* in which the participant saw that they had not correctly predicted the outcome), and Δ *neural activity goal-no goal*_LP-NLP_ (*video clips* showing player LP converting a shot into a goal contrasted with *video clips* showing player LP executing the shot but not scoring a goal minus *video clips* showing player NLP converting a shot into a goal contrasted with *video clips* showing player NLP executing the shot but not scoring a goal). To correct for the fact that player LP executed more shots than player NLP (Table S1), only parameter estimates for *video clips* in which LP’s start position was on the left were used for these contrasts (i.e. 32 trials in which player LP executed the shot and 32 trials in which player NLP executed the shot). Because the participants’ individual response selection did not always result in a completely balanced two-by-two-by-two design (Table S1) individual parameter estimates were first averaged separately for each cell and then averaged across cells. Importantly, the participants’ trial-by-trial correctness in predicting the players’ behavior (*own reward*) and each player’s trial-by-trial success in scoring a goal (*empathic reward*) were not related (player LP, mean r = 0.02 +/− 0.02 [mean +/−  s.e.m., backtransformed mean/s.e.m. of Fisher transformed trial-by-trial correlation coefficients], T[23] = 1.0, p > 0.200; player NLP, r = 0.01 +/− 0.04, T[23] = 0.4; p > 0.200 two-sided), thus no correction was necessary. Individual contrast images were spatially smoothed (8 mm isotropic Gaussian kernel) for further analyses.

Statistical significance of predicted effects was assessed at random effects group level, following a two-step procedure. First, we performed a whole-brain analysis to identify regions in the VS/mOFC^14,15^ that showed stronger neural activity when participants saw that they had correctly predicted the outcome of a trial than when they saw that they had not correctly predicted the outcome of a trial (contrast Δ *neural activity correct-incorrect response*). Next, to test whether in these regions neural responses to a player’s success would be modulated by behavioural similarity, we placed three spherical ROIs (6 mm radius) at the most significantly activated voxel in the left/right ventral striatum and mOFC, respectively, and averaged voxel-wise contrast estimates Δ *neural activity goal-no goal*_LP-NLP_ across all voxels in each ROI. These mean contrast estimates were used for univariate correlation analyses with Δ *similarity*_LP-NLP_. This two-step approach avoided overestimation of effect sizes that might occur in mass univariate analyses^42^. For completeness, we also report coordinates and statistical values for the highest activated voxel within each ROI in corresponding voxel-wise analyses (Fig. S3 in the Supplemental Material).

Finally, to identify brain regions outside the VS/mOFC that might show phenotype-dependent modulation of neural activity, we performed a whole-brain correlation analysis between Δ *similarity*_LP-NLP_ and voxel-wise contrast estimates Δ *neural activity goal-no goal*_LP-NLP_.

### Statistical inference

Student’s T statistics were used for all tests. Degrees of freedom (df) for each test are given in square brackets. Pearson’s product-moment correlation coefficient was used to estimate correlation strengths. Behavioural effects and effects based on mean contrast estimates for ROIs were tested two-sided, and voxel-wise effects were tested one-sided. For the whole brain analyses, statistical maps were thresholded at a voxel-wise height threshold of p = 0.001 and effects were assessed at cluster level, correcting for family-wise errors [FWE] according to Random Field Theory^43^. In the voxel-wise analyses within ROIs (Fig. S3) effects in individual voxels were assessed using small-volume FWE correction according to Random Field Theory^43^. Effects were considered significant if the probability of false positives (p) did not exceed α = 0.050. Exact p values are given for p > 0.001 and p < 0.200.

## Supplementary information

Supplemental material.

## Data Availability

The datasets generated and analyzed during this study are available from the corresponding author on reasonable request.
