# Supplementary material for: Empathic responses to unknown others are modulated by shared behavioural traits
Source: Sci Rep. 2020 Feb 6;10:1938. doi: 10.1038/s41598-020-57711-6 (PMC7005154; doi:10.1038/s41598-020-57711-6)
Supplement: Supplementary file 1 — Supplemental material. [file 41598_2020_57711_MOESM1_ESM.doc]

**Supplemental Information**

**Empathic responses to unknown others are modulated by shared behavioural traits**

Silke Anders^1*^, Christian Beck^1^, Martin Domin^2^ und Martin Lotze^2^

^1^Social and Affective Neuroscience, Department of Neurology, Universität zu Lübeck, Lübeck, Germany

^2^Functional Imaging Unit, Center for Diagnostic Radiology, University Medicine Greifswald, Greifswald, Germany

| **Start position** | **Shooter** | **Block direction** | **Percent of trials** | **Outcome** | **Participants’ response** | **Correctness of response** | **Percent of trials** |
| --- | --- | --- | --- | --- | --- | --- | --- |
| LP left – NLP right | LP | left | 12.5 % | goal | goal | correct | 6.3 % |
|  |  |  |  |  | no goal | not correct | 6.2 % |
|  |  | right | 12.5 % | no goal | no goal | correct | 6.7 % |
|  |  |  |  |  | goal | not correct | 5.8 % |
|  | NLP | right | 12.5 % | goal | goal | correct | 5.6 % |
|  |  |  |  |  | no goal | not correct | 6.9 % |
|  |  | left | 12.5 % | no goal | no goal | correct | 5.4 % |
|  |  |  |  |  | goal | not correct | 7.1 % |
| NLP left – LP right | LP | right | 25 % | goal | goal | correct | 18.0 % |
|  |  |  |  |  | no goal | not correct | 7.0 % |
|  |  | left | 25 % | no goal | no goal | correct | 17.0% |
|  |  |  |  |  | goal | not correct | 8.0 % |
| **Supplemental Table S1. Overview of trial types and mean correctness of the participants’ responses for each trial type.** The robots assigned to player LP and NLP were distinguished by the colour of their eyes and chest marks (orange or green), counterbalanced across participants. Only trials with start position LP left – NLP right were included in analyses of MRT data. Trial outcome and correctness of responses were not correlated (see Results in main text). | | | | | | | |

**
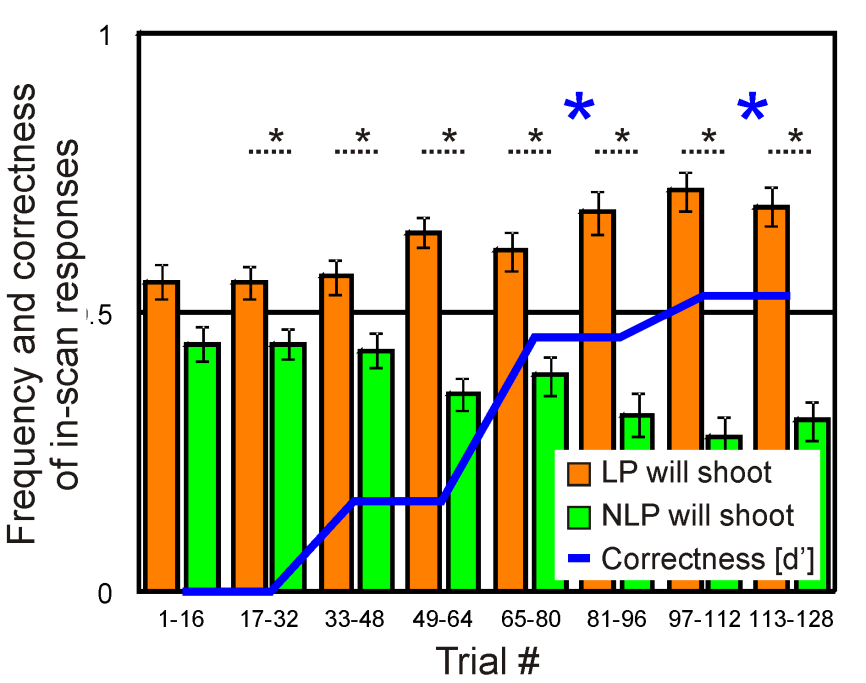
**

**Supplemental Figure S1. Frequency distribution and correctness of in-scan responses across trials.** Participants selected responses that were compatible with the prediction that player LP would execute the shot significantly more often than responses that were compatible with the prediction that player NLP would execute the shot in all but the very first 16-trial blocks (trial 17-128). In contrast, responses that correctly predicted who would execute the shot (implying that participants correctly used the rule to predict the players’ behaviour) were selected significantly more often than responses that did not correctly predict the shooter only in the last four 16-trials blocks (trial 65-128). Error bars represent s.e.m. Asterisks indicate significant effects (p < .05, two-sided), black asterisk correspond to response frequencies, blue asterisks correspond to correctness of response (d’).

| **Contrast** | **Region** | **T-value**  **at peak(s)** | **Coordinates at peak(s)** | **Cluster size** | ***p*** |
| --- | --- | --- | --- | --- | --- |
| *Own reward*  (correct response minus incorrect response) | Bilateral ventral striatum | T = 10.7  T = 9.5 | [-18, 9, -12]  [15, 15, -3] | 1069 | p < .001 |
|  | mOFC | T = 5.3 | [6, 51, -6] | 313 | p < .001 |
|  | Left occipital cortex | T = 5.2 | [ -15, -102, 3] | 350 | p < .001 |
|  | Right occipital cortex | T= 6.2 | [33, -93, -6] | 292 | p < .001 |
| *Similarity x empathic reward*  (*similarity* _LP-NLP_ x  *goal-no goal* _LP-NLP_) | vACC | T = 5.3 | [ -12, 48, 3] | 109 | p = .005 |
| **Supplemental Table S2. Summary of significant clusters in the whole-brain analyses.** Cluster sizes are given in number of voxels (3 x 3 x 3 mm³). All p-values are FWE-corrected at cluster level according to Random Field theory (see main text). MOFC, medial orbitofrontal cortex, vACC, ventral anterior cingulate cortex. | | | | | |

**
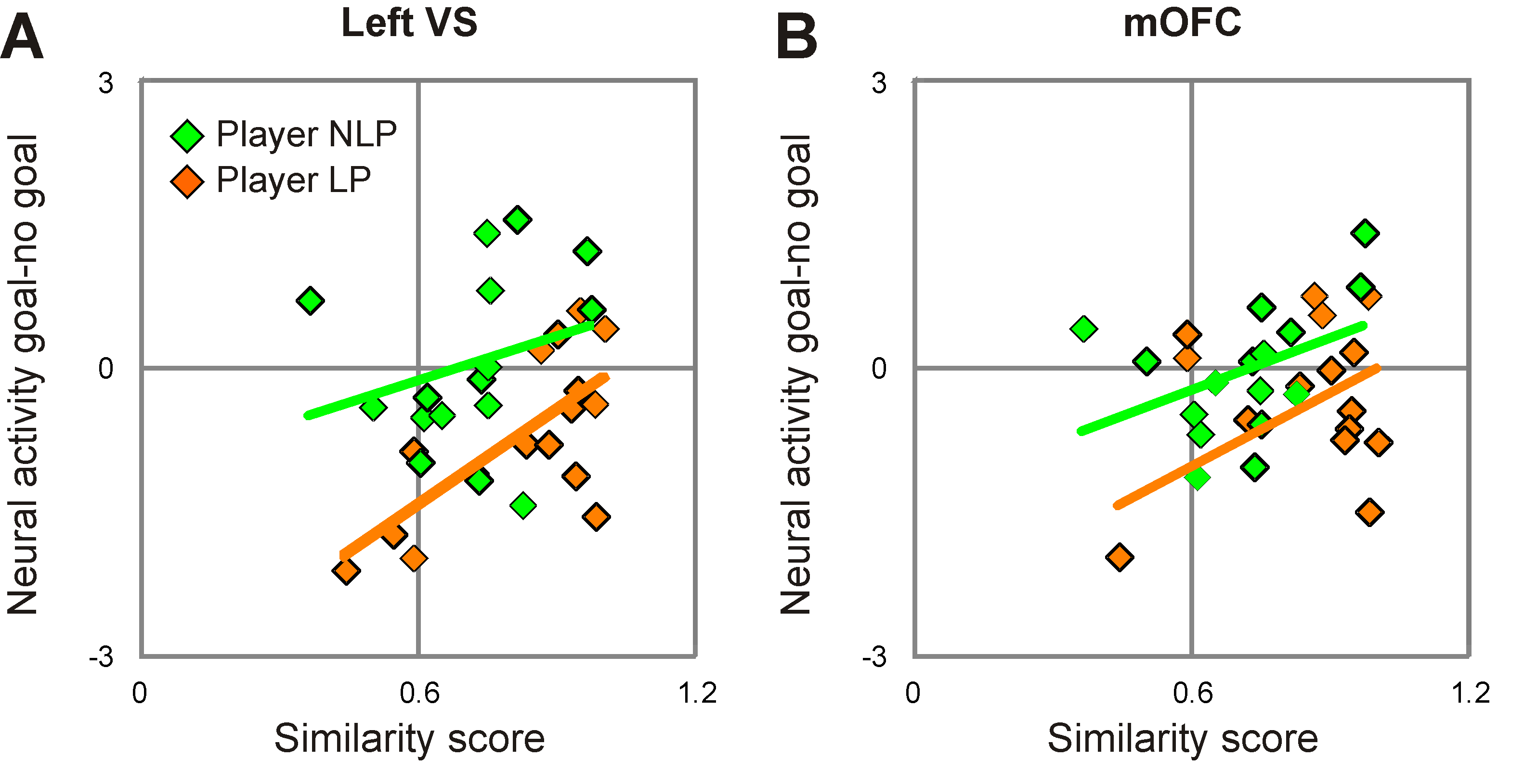
**

**Supplemental Figure S2. Correlation between behavioural similarity and empathic-reward related neural activity for each player in the left VS and mOFC.** Each dot denotes a participant. In the left VS, the correlation was significant for player LP, but not for player NP (*similarity score* x *neural activity goal-no goal*, player LP, r = .70; T[14] = 3.7; p = .001; player NLP, r = .26; T[14] = 1.0; p = .167). In the mOFC, the correlation was just below statistical significance for both player LP and player NLP (*similarity score* x *neural activity goal-no goal*, player LP, r = .41; T[14] = 1.7; p = .056; player NLP, r = .41; T[14] = 1.7; p = .056; all p one-sided). VS, ventral striatum; mOFC, medial orbitofrontal cortex.

**
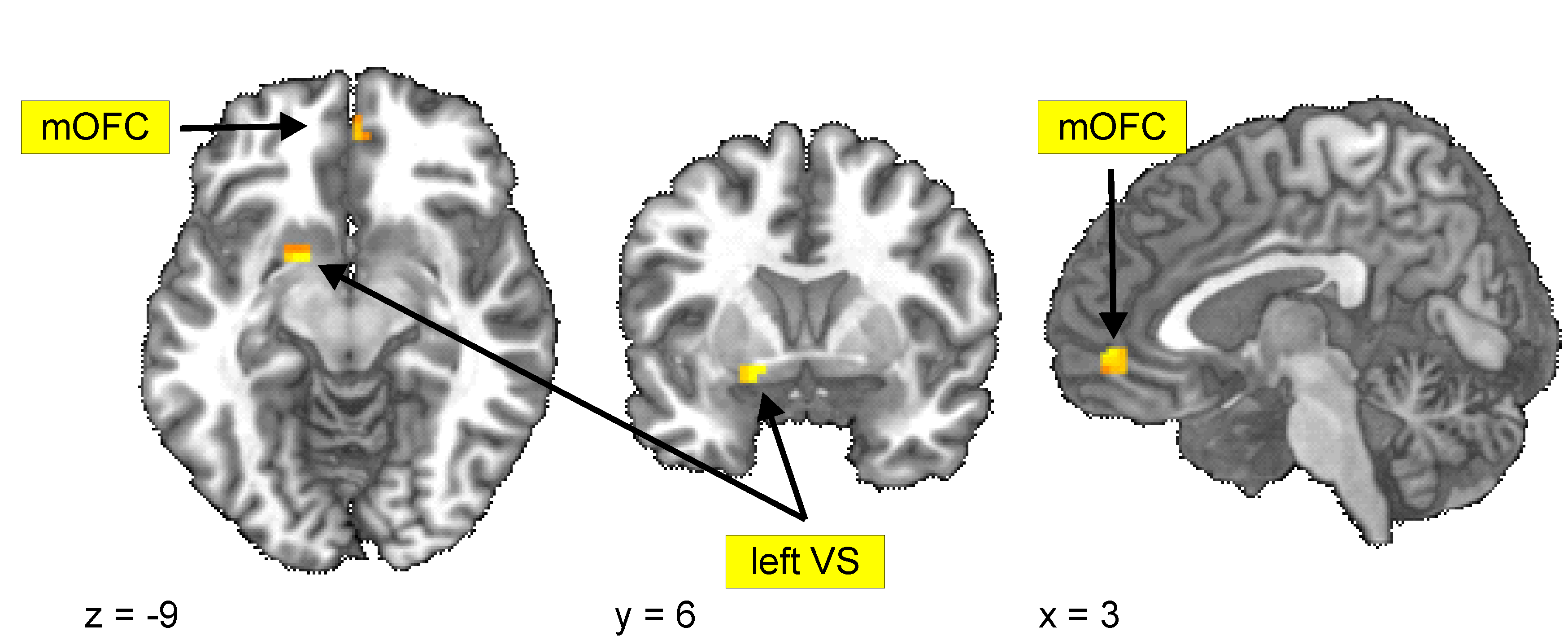
**

**Supplemental Figure S3. Voxel-wise correlation analysis of differences in behavioural similarity and differences in empathic-reward related neural activity within the left VS and mOFC ROIs.** For visualization, statistical parametric maps (masked with two 6 mm spheres, centred at x = -18, y = 9, z = -12 and x = 6, y = 51, z = -6, respectively) are thresholded at a voxel-wise height threshold of p = .05 and superimposed onto sections of a T1-weighted map of a standard brain (MNI). Coordinates and statistical values of the most significant voxel (contrast Δ *similarity* _LP-NLP_ x Δ *neural activity* *goal-no goal* _LP-NLP_) are x = -15, y = 6, z = -9, T[22] = 3.1, p = .036 (left VS) and x = 3, y = 54, z = -3, T[22] = 3.2, p = .031 (mOFC); p are small-volume FWE-corrected. VS, ventral striatum; mOFC, medial orbitofrontal cortex.

**Supplemental study**

Twelve volunteers (6 women, mean age 21.8 years, range 19-26 years; 6 men, mean age 23.0 years, range 21-30 years; all with no record of neurological or psychiatric disorders), recruited at Universität zu Lübeck, Germany, participated in a supplemental study conducted to confirm the external validity of the similarity score used in the main study.

Participants (i) completed the self-report questionnaire used in the main study (i.e. the *Assertiveness* and *Dominance* scales of the *International Personality Item Pool*, IPIP), (ii) interacted with five other participants, (iii) rated how similar each of the five other participants was to themselves on a self-similarity questionnaire not used in the main study, and (iv) rated each of the five other participant’s tendency to lead on the questionnaire used in the main study (assertiveness-influence-aggressiveness scales).

During the interaction sessions, participants (6 women in session 1, 6 men in session 2) solved tasks in changing teams of three. Tasks included quizzes relating to general knowledge and specific knowledge about Lübeck and pantomiming or sketching a given term to the other team. After each round (comprising two pantomime tasks, two sketching tasks and two quizzes, totalling approximately 15 min per round), teams were shuffled such that after ten rounds participants had played once in all possible combinations of team members.

The self-similarity questionnaire not used in the main study asked participants (raters) to decide for each of the five other participants (targets) in their session whether the target was more similar to the rater or to a given other participant. For example, for participant A, the self-similarity questionnaire comprised 20 questions in the form of “*Is participant B more similar to you or to participant C*?” Each questionnaire comprised all possible 5 x 4 = 20 pairwise comparisons. Data for each rater were reduced by counting the number of instances the rater judged a given target to be more similar to the rater than to the other participant. This resulted in 6 x 5 = 30 self-similarity scores per session (e.g. A-B, A-C, A-D, A-E, A-F, B-A, B-C, ..., B-F, ...., E-F).

To examine whether the computed similarity score used in the main study (i.e. [1 – |*tendency-to-lead* [IPIP] _participant_ - *tendency-to-lead* [assertiveness-influence-aggressiveness scale] _other participant_|]) provides a valid estimate of perceived self-similarity, we first tested whether the participants’ self-reported tendency to lead on the IPIP correlated with average tendency-to-lead ratings on the assertiveness-influence-aggressiveness scale by other participants. This was the case (Pearson’s r = .54, T[10] = 2.0, p = .035, one-sided, **Figure S4 A**). This suggests that the IPIP and the assertiveness-influence-aggressiveness scales measure similar things. Next, we tested whether computed similarity scores predicted self-similarity ratings. This was the case both within and across session (women, Pearson’s r = .38, T[28] = 2.2, p = .019, **Figure S4 B**; men, Pearson’s r = .41, T[28] = 2.4, p = .008, **Figure S4 C;** all participants, Pearson’s r = .41, T[58] = 3.4, p < .001, all one-sided).

The results of the supplemental study show that (i) the IPIP questionnaire and the assertiveness-influence-aggressiveness scale measure similar things, (ii) when people assess how similar another person is to themselves a person’s tendency to lead is an important factor (note that self-similarity ratings were obtained before participants rated their fellow participants’ tendency to lead and a couple of days after participants completed the IPIP as part of the recruitment process, so participants were not primed to focus on tendency to lead when judging self-similarity) and (iii) the computed similarity score used in the main study provides a valid estimate of perceived self-similarity.


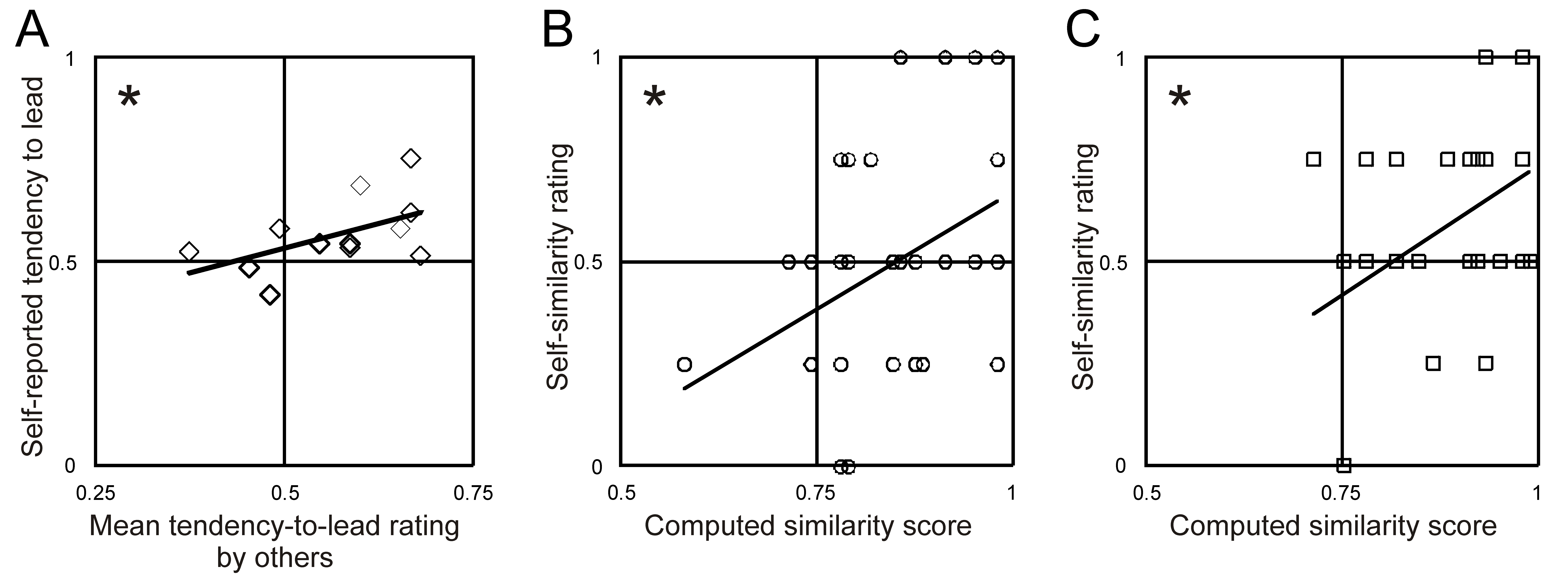


**Supplemental Figure S4. A**, Correlation between the participants’ self-reported tendency to lead (on the IPIP) and average tendency-to-lead ratings by others (on the assertiveness-influence-aggressiveness scales). **B, C** Correlation between the participants’ self-similarity ratings and similarity scores as computed in the main study (B, women; C, men). Asterisks indicate significant correlations.
